# Characteristics of menstrual disorders and reproductive hormones in women with epilepsy at an Indonesian national referral hospital

**DOI:** 10.3389/fneur.2022.964761

**Published:** 2022-09-20

**Authors:** Fitri Octaviana, Kanadi Sumapraja, Winnugroho Wiratman, Luh Ari Indrawati, Astri Budikayanti

**Affiliations:** ^1^Department of Neurology, Faculty of Medicine, Universitas Indonesia, Jakarta, Indonesia; ^2^Department of Neurology, Dr. Cipto Mangunkusumo National Referral Hospital, Jakarta, Indonesia; ^3^Department of Obstetrics and Gynecology, Faculty of Medicine, Universitas Indonesia, Jakarta, Indonesia; ^4^Department of Obstetrics and Gynecology, Dr. Cipto Mangunkusumo National Referral Hospital, Jakarta, Indonesia

**Keywords:** menstrual disorders, epilepsy, women, reproductive hormones, dysmenorrhea

## Abstract

**Objective:**

Menstrual disorders are more common in women with epilepsy than in those without epilepsy. This study aimed to examine the characteristics of reproductive function in women with epilepsy at an Indonesian national referral hospital.

**Methods:**

A case-control study was conducted from March 2020 to March 2021. Women with and without epilepsy aged ≥18 years were enrolled. All women were premenopausal before epilepsy diagnosis. Data on demographic characteristics, menstrual profiles, epileptic syndrome, seizure type, seizure frequency, etiology, localization, and anticonvulsant medication were collected. Hormone levels (follicle stimulating hormone, luteinizing hormone, prolactin, and estradiol) were measured.

**Results:**

A total of 72 women with and 50 without epilepsy (controls) were included. Dysmenorrhea was more common in women with epilepsy than in those without (59.7 vs. 20%, *p* < 0.001; odds ratio: 5.931 [95% confidence interval: 2.566–13.709]). Marriage rates were higher in women without epilepsy (82 vs. 45.8%, *p* < 0.001). No difference was found in hormone levels between the groups. The frequency of seizures was associated with prolactin and estradiol levels (*p* < 0.001). Polytherapy with clobazam was associated with menstrual cycle regularity. In women with epilepsy with menstrual disorders, valproic acid was associated with higher estradiol levels (*p* = 0.001) and lamotrigine with lower follicle stimulating hormone levels (*p* = 0.008).

**Significance:**

Women with epilepsy experienced more dysmenorrhea. A higher frequency of seizures associated with lower prolactin and estradiol levels. Polytherapy with clobazam was associated with irregular menstrual cycles, while valproic acid and lamotrigine was associated with estradiol and follicle stimulating hormone levels.

## Introduction

Epilepsy is a neurological disorder that commonly affects women, with an estimated prevalence of 6.85 cases per 1,000 population (1, 2) and range of ~0.3–0.7% in women of childbearing age (3, 4). Approximately 1.5 million women with epilepsy (WWE) in the United States and 1.3 million WWE in India belong to the reproductive age group (5, 6). Epilepsy is considered an inherited disease by certain inhabitants, thus influencing certain people to hesitate marrying WWE. A study in India, which is similar to Indonesia in social and economic aspects, revealed that marriage rates among WWE in India were far lower than those among women without epilepsy (WWoE) (7). However, confirmed data in Indonesia are still lacking. Stigma surrounding epilepsy in Indonesia remains profound (8).

Epilepsy and reproductive hormones have a complex bidirectional relationship, and the reproductive health of WWE requires special consideration, especially in patients who have been using anti-seizure medications (ASMs) for a long period of time (9). Reproductive hormones, such as follicle stimulating hormone (FSH), luteinizing hormone (LH), progesterone, and estradiol, are important determinants of female reproductive function. These hormones affect oocyte maturation, ovulation, and menstruation. A previous study demonstrated that hormonal changes in WWE affect neuronal activity and increase the risk of worsening seizures; for example, estrogen has an excitatory effect on neurons that can generate seizures (10). Epilepsy can also affect reproductive endocrine function through the hypothalamic-pituitary-adrenal (HPA) axis (11). Several studies have shown an increase in the frequency of menstrual disorders, fertility problems, and hormonal dysfunction in WWE. However, fluctuating hormonal levels can also affect the frequency of seizures and metabolism of ASMs and vice versa (2). In one study, menstrual disorders were reported in one in three WWE compared to one in seven women in the general population (12).

The increasing prevalence of menstrual disorders in WWE can also be attributed to the side effects of certain ASMs. Cytochrome P450 inducers, such as carbamazepine and phenytoin, can affect circulating hormone levels, while continued use of clonazepam can affect menstrual cycle regularity (9). Other ASMs such as valproic acid (VPA) can also cause side effects, such as polycystic ovaries and hyperandrogenism (13). The relationship between epilepsy and menstrual disorders remains unclear. The purpose of this study was to determine the characteristics of reproductive function in WWE, with a focus on dysmenorrhea and menstrual disorders, and to evaluate differences in reproductive hormone levels between WWE and WWoE.

## Materials and methods

### Study design and participants

This study was approved by the Faculty of Medicine Universitas Indonesia Ethics Committee and Cipto Mangunkusumo Hospital research section (ethical review number: 1393/UN2.F1/ETIK/PPM.00.02/2019). We conducted a case-control study from March 2020 to March 2021 at Dr. Cipto Mangunkusumo National Referral Hospital, Jakarta. All patients provided written informed consent. The subjects were recruited using convenient consecutive sampling method. The sample size was determined using the calculation for 2 groups with a 95% confidence interval (95% CI) and power of 80%, using the prevalence of menstrual disorders in WWE and WWoE based on previous study (7). The inclusion criteria for the case group (WWE) and control group (WWoE) were those aged ≥18 years and already had their menstrual cycle. The exclusion criteria for the case group were WWE who had already had menopause before being diagnosed epilepsy, while the exclusion criteria for control group were WWoE who already had menopause and had comorbidities such as autoimmune disease and other chronic diseases. Subjects in the control group who had menstrual disorders were recruited from patients who visited the gynecology outpatient clinic at Dr Cipto Mangunkusumo National Referral Hospital, while subjects in the control group without menstrual disorders were recruited from patients who visited general outpatient clinics at the same hospital and did not have any symptoms of menstrual disorders. Thereafter, WWE and WWoE with menstrual disorders were compared, and a similar comparison was made between WWE and WWoE without menstrual disorders.

Demographic and clinical data were obtained primarily through interviews and secondarily through medical records. Blood samples were subsequently taken from both groups after the interview to measure levels of reproductive hormones which were FSH, LH, prolactin, and estradiol. The confounder factor was the blood samples were taken randomly at unspecified times without regard to phases of the menstrual cycle in both groups.

### Data collection

Independent variables comprised the subjects' age, onset of epilepsy, duration of epilepsy, seizure type and frequency, type and number of ASMs consumed, history of status epilepticus, etiology of epilepsy, localization of epilepsy, age of menarche, and marital history. The classification of epilepsy types and etiology were defined based on the International League Against Epilepsy 2017 classification guidelines (14). To determine structural etiology and localization of epilepsy, EEG and brain MRI were performed on all WWE. All patients on ASM polytherapy were further analyzed to determine the association between ASM combinations and menstrual disorders or dysmenorrhea. ASMs were further categorized further into the following three categories: (a) inducers, which included carbamazepine, phenytoin, and phenobarbital; (b) inhibitors, which included VPA; and (c) miscellaneous for the remaining types of ASMs. Subjects with controlled seizures were defined as those who had been seizure free throughout the preceding year.

Dependent variables included menstrual disorders and dysmenorrhea. Menstrual disorders were defined as any deviation from the normal menstrual cycle based on the 2011 International Federation of Gynecology and Obstetrics guidelines (15). In this study, we classified menstrual disorders based on frequency and regularity. Menstrual cycles were considered of normal frequency if they were within the range of 21–35 days and abnormal if they were outside that range. Regularity was classified as normal if the variation between cycles was limited to 2–20 days over a 12-months period. Dysmenorrhea was defined as discomfort and pain during the menstrual period (16).

### Statistical analysis

Data were analyzed using SPSS version 20.0. Data on subject characteristics measured in categorical form are presented in proportions. Data in numerical form were analyzed using the *Kolmogorov–Smirnov* test to determine the distribution of the data. Normally distributed data were interpreted using a *p*-value > 0.05 to indicate statistical significance and are presented as the mean and standard deviation (SD), while abnormally distributed data were interpreted using a *p*-value < 0.05 to indicate statistical significance and are presented as the median and range. To determine the potential factors associated with menstrual disorders and dysmenorrhea, an initial bivariate analysis was conducted to determine the factors that had a statistically significant effect. The chi-square test or Fisher's exact test was used for categorical variables, and the student's *t*-test or Mann–Whitney test was used for numerical variables.

## Results

### Demographic and clinical characteristics of WWE and WWoE

Seventy-two WWE and 50 WWoE participated in this study. There were no missing data in this study. The demographic characteristics of WWE compared to those of WWoE are displayed in Table 1. The mean age of WWE was younger than that of WWoE (*p* < 0.05). The age of menarche was comparable between the groups (~12 years). The numbers of WWE (case) and WWoE (control) who had menstrual disorders were similar (30 and 32, respectively). However, the number of WWE was higher than that of WWoE in the normal menses group (42 and 18, respectively). Dysmenorrhea was more common in WWE than in WWoE (59.7 vs. 20%; odds ratio [OR]: 5.931, 95% CI: 2.566–13.709). WWE were less likely to be married than WWoE (45.8 vs. 82%; *p* < 0.001). Most WWE were aged ≤ 40 years (83.3%), and the majority (73.6%) were aged >10 years at the time of epilepsy onset. The most common type of seizure encountered was the focal type (95.8%), with most subjects claiming to have uncontrolled seizures (63.9%). Only eight patients (11.1%) had a history of status epilepticus in their lifetime. Most WWE reported one or two seizures per month (76.1%).

**Table 1:** Demographic characteristics of women with epilepsy and women without epilepsy.

**Variable**	**Women with epilepsy (*n* = 72)**	**Women without epilepsy (*n* = 50)**	***P*-value**	**OR (95% CI)**
	***N* (%)**	***N* (%)**		
Age (years) [Mean (SD)]	29.4 (9.1)	34.38 (7.5)	**0.002**	
Age at menarche (years) [Mean (SD)]	12.85 (1.4)	12.86 (1.3)	0.959	
Age (years)				
≤ 40 years old	60 (83.3)	39 (78)	0.488	0.709 (0.285–1.765)
>40 years old	12 (16.7)	11 (22)		
Age of menarche			1.000	1.091 (0.354–3.363)
<12 years old	8 (11.1)	6 (12)		
≥12 years old	64 (88.9)	44 (88)		
Menstrual disorders				
Normal	42 (58.3)	18 (36)		
Abnormal	30 (41.7)	32 (64)	**0.018**	**0.402 (0.191–0.845)**
- Frequency	12 /30 (40)	7 /32 (21.9)		
- Regularity	18 /30 (60)	25 /32 (78.1)		
Dysmenorrhea				
Yes	43 (59.7)	10 (20)	**0.000**	**5.931 (2.566–13.709)**
No	29 (40.3)	40 (80)		
Marital status				
Married	33 (45.8)	41 (82)	**0.000**	**0.186 (0.079–0.438)**
Unmarried	39 (54.2)	9 (18)		
Age at onset of epilepsy				
≤ 10 years old	19 (26.4)			
>10 years old	53 (73.6)			
Duration of epilepsy (years) [Median (min–max)]	7.5 (0–38)			
Seizure type				
Focal	69 (95.8)			
Generalized	3 (4.2)			
Location of epilepsy focus				
Temporal	36 (50)			
Extratemporal	36 (50)			
Controlled seizure				
Yes	26 (36.1)			
No	46 (63.9)			
Frequency of seizures (months)				
1	24 (52.2)			
2	11 (23.9)			
3	4 (8.7)			
4	3 (6.5)			
5	2 (4.3)			
8	1 (2.2)			
25	1 (2.2%)			
History of status epilepticus (Lifetime)				
Yes	8 (11.1)			
No	64 (88.9)			
Etiology of epilepsy				
Structural	28 (38.9)			
Infection	6 (8.3)			
Immune	6 (8.3)			
Unknown	31 (43.1)			
Genetic	1 (1.4)			
Number of ASMs				
Monotherapy	34 (47.2)			
Polytherapy	38 (52.8)			

Student's t-test showed that age between the two groups was significantly different, while the chi-square test revealed that menstrual disorders, dysmenorrhea, and marital status differed significantly between the two groups. SD, standard deviation; OR, odds ratio; 95% CI, 95% confidence interval; ASM, anti-seizure medication. The bold values indicates the p value is significant (<0.05).

The analysis was performed separately between groups with or without menstrual disorders, as shown in Table 2. The demographic characteristics of the subjects based on the presence of menstrual disorders are displayed in Table 2. In the group with menstrual disorders, there were 30 WWE and 32 WWoE. Most WWE and WWoE with menstrual disorders were aged ≤ 40 years (80 and 84.4%, respectively), and the age at onset of menarche was ≥12 years in most patients (83.3 and 90.6%, respectively). Dysmenorrhea was significantly more common in WWE than in WWoE in the menstrual disorders group (56.7 vs. 9.4%; OR: 12.64, CI: 3.146–50.794). In the group without menstrual disorders, there were 42 WWE and 18 WWoE. The proportion of WWE who had dysmenorrhea was higher than that of WWoE (61.9 and 38.9%, respectively); however, the difference was not statistically significant.

**Table 2:** Comparison of demographic and hormone characteristics between women with menstrual disorders and women without menstrual disorders.

	**Variable**	**Women with epilepsy**	**Women without epilepsy**	***P*-value**	**OR (95% CI)**
		***N* (%)**	***N* (%)**		
Women with menstrual disorders	Age (years)				
	≤ 40 years old	24 (80%)	27 (84.4%)	0.746	1.35 (0.365–4.993)
	>40 years old	6 (20%)	5 (15.6%)		
	Age (years) [mean (SD)]	30.27 (9.5)	32.81 (8.0)	0.257	
	Age of menarche				
	<12 years old	5 (16.7%)	3 (9.4%)	0.467	0.517 (0.112–2.384)
	≥12 years old	25 (83.3%)	29 (90.6%)		
	Dysmenorrhea				
	Yes	17 (56.7%)	3 (9.4%)	**0.000**	**12.64 (3.146–50.794)**
	No	13 (43.3%)	29 (90.6%)		
	Marital status				
	Married	12 (40%)	23 (71.9%)	**0.02**	**3.833 (1.326–11.084)**
	Unmarried	18 (60%)	9 (28.1%)		
Women without menstrual disorders	Age (years)				
	≤ 40 years old	36 (85.7%)	12 (66.7%)	0.156	0.333 (0.09–1.231)
	>40 years old	6 (14.3%)	6 (33.3%)		
	Age (years) [mean (SD)]	28.79 (8.9)	37.17 (5.8)	**0.001**	
	Age of menarche				
	<12 years old	3 (7.1%)	3 (16.7%)	0.352	2.6 (0.471–14.339)
	≥12 years old	39 (92.9%)	15 (83.3%)		
	Dysmenorrhea				
	Yes	26 (61.9%)	7 (38.9%)	0.156	2.554 (0.822–7.936)
	No	16 (38.1%)	11 (61.1%)		
	Marital status				
	Married	21 (50%)	18 (100%)	**0.000**	**0.538 (0.403–0.72)**
	Unmarried	21 (50%)	0		

The chi-square test revealed that in the group with menstrual disorders, dysmenorrhea differed significantly between women with epilepsy and those without, while marital status was also significantly different in the group without menstrual disorders. SD, standard deviation; OR, odds ratio; 95% CI, 95% confidence interval. The bold values indicates the p value is significant (<0.05).

### Factors associated with dysmenorrhea and menstrual disorders in WWE

Table 3 details the association of dysmenorrhea and menstrual disorders in WWE. No factors were related to the incidence of dysmenorrhea or menstrual disorders in WWE. However, clobazam was significantly associated with menstrual disorders (*p* = 0.033). All patients who used clobazam were on polytherapy.

**Table 3:** Crosstabulation of dysmenorrhea and menstrual disorders in women with epilepsy.

**Variable**	**Women with epilepsy (*****N*** = **72)**							
	**Dysmenorrhea**				**Menstrual disorders**			
	**Yes (*N* = 43)**	**No (*N* = 29)**	***P*-value**	**OR (95% CI)**	**Yes (*N* = 30)**	**No (*N* = 42)**	***P*-value**	**OR (95% CI)**
Age (years)								
≤ 40 years old	37 (86%)	23 (79.3%)	0.527	0.62 (0.18–2.16)	24 (80%)	36 (85.7%)	0.539	1.5 (0.43–5.204)
>40 years old	6 (14%)	6 (20.7%)			6 (20%)	6 (14.3%)		
Age at onset of epilepsy								
≤ 10 years old	9 (20.9%)	10 (34.5%)	0.276	1.99 (0.69–5.75)	7 (23.3%)	12 (28.6%)	0.787	1.31 (0.45–3.87)
>10 years old	34 (79.1%)	19 (65.5%)			23 (76.7%)	30 (71.4%)		
Duration of epilepsy (years) [median (min–max)]	7 (0-36)	11 (0-38)	0.154		7.5 (0-38)	7.5 (0-35)	0.693	
Seizure type								
Focal	41 (95.3%)	28 (96.6%)	0.646	1.37 (0.12–15.8)	27 (90%)	42 (100%)	0.068	0.39 (0.29–0.52)
Generalized	2 (4.7%)	1 (3.4%)			3 (10%)	0 (0%)		
Location of epilepsy focus								
Temporal	22 (51.2%)	14 (48.3%)	0.810	0.89 (0.35–2.29)	13 (43.3%)	23 (54.8%)	0.339	1.58 (0.61–4.06)
Extratemporal	21 (48.8%)	15 (51.7%)			17 (56.7%)	19 (45.2%)		
Controlled seizure								
Yes	18 (41.9%)	8 (27.6%)	0.317	0.53 (0.19–1.46)	12 (40%)	14 (33.3%)	0.623	0.75 (0.28–1.98)
No	25 (58.1%)	21 (72.4%)			18 (60%)	28 (66.7%)		
History of status epilepticus								
Yes	6 (14%)	2 (6.9%)	0.461	0.46 (0.09–2.44)	5 (16.7%)	3 (7.1%)	0.265	0.39 (0.084–1.8)
No	37 (86%)	27 (93.1%)			25 (83.3%)	39 (92.9%)		
Etiology of epilepsy								
Structural	14 (32.6%)	14 (48.3%)			14 (46.7%)	14 (33.3%)		
Infection	3 (7%)	3 (10.3%)	0.203		2 (6.7%)	4 (9.5%)	0.411	
Immune	5 (11.6%)	1 (3.4%)			2 (6.7%)	4 (9.5%)		
Genetic	1 (2.3%)	0 (0%)			0 (0%)	1 (2.4%)		
Unknown	20 (46.5%)	11 (37.9%)			12 (40%)	19 (45.2%)		
Epilepsy syndrome								
Symptomatic/cryptogenic	38 (88.4%)	27 (93.1%)	0.407	1.78 (0.32–9.84)	28 (93.3%)	37 (88.1%)	0.376	0.52 (0.09–2.93)
Idiopathic	5 (11.6%)	2 (6.9%)			2 (6.7%)	5 (11.9%)		
Number of ASM								
Monotherapy	20 (46.5%)	14 (48.3%)	1.000	1.07 (0.42–2.76)	14 (46.7%)	20 (47.6%)	1.000	1.04 (0.4–2.66)
Polytherapy	23 (53.5%)	15 (51.7%)			16 (53.3%)	22 (52.4%)		
Age of menarche								
<12 years old	4 (9.3%)	4 (13.8%)	0.706	1.56 (0.36–6.81)	5 (16.7%)	3 (7.1%)	0.265	0.39 (0.08–1.75)
≥12 years old	39 (90.7%)	25 (86.2%)			25 (83.3%)	39 (92.9)		
Marital status								
Married	18 (41.9%)	15 (51.7%)	0.474	1.49 (0.58–3.84)	12 (40%)	21 (50%)	0.475	1.5 (0.58–3.87)
Unmarried	25 (58.1%)	14 (48.3%)			18 (60%)	21 (50%)		
Types of ASM (as monotherapy or polytherapy)								
Phenytoin (*n* = 20)	14/20	6/20	0.298	0.54 (0.2–1.6)	7/20	13/20	0.478	0.679 (0.23–1.98)
Carbamazepine (*n* = 22)	12/22	10/22	0.608	1.36 (0.5–3.8)	9/22	13/22	0.931	0.96 (0.35–2.65)
Valproic acid (*n* = 17)	13/17	4/17	0.158	0.37 (0.1–1.3)	7/17	10/17	0.963	0.97 (0.32–2.94)
Levetiracetam (*n* = 24)	11/24	13/24	0.126	2.36 (0.9–6.4)	10/24	14/24	1.000	1 (0.37–2.7)
Clobazam (*n* = 13)	7/13	6/13	0.757	1.34 (0.4–4.5)	9/13	4/13	**0.033**	**4.07 (1.12–14.83)**
Lamotrigine (*n* = 16)	12/16	4/16	0.248	0.41 (0.1–1.4)	7/16	9/16	0.848	1.12 (0.36–3.43)
Topiramate (*n* = 15)	7/15	8/15	0.375	1.96 (0.6–6.2)	7/15	8/15	0.659	1.29 (0.41–4.06)
Clonazepam (*n* = 2)	1/2	1/2	1.000	1.5 (0.1–25)	0/2	2/2	0.507	1.75 (1.43–2.14)
Types of ASM (as monotherapy)								
Phenytoin	4 (20%)	2 (14.3%)	1.000		2 (14.2%)	4 (20%)	1.000	
Carbamazepine	3 (15%)	2 (14.3%)	1.000		2 (14.2%)	3 (15%)	1.000	
Valproic acid	2 (10%)	1 (7.1%)	1.000		0 (0.0%)	3 (15%)	0.251	
Levetiracetam	5 (25%)	6 (42.9%)	0.458		5 (35.7%)	6 (30%)	1.000	
Clobazam	0 (0.0%)	1 (7.1%)	0.412		1 (7.1%)	0 (0.0%)	0.412	
Lamotrigine	4 (20%)	1 (7.1%)	0.379		2 (14.2%)	3 (15%)	1.000	
Topiramate	2 (10%)	1 (7.1%)	1.000		2 (14.2%)	1 (5%)	0.555	
Clonazepam	0 (0.0%)	0 (0.0%)			0 (0.0%)	0 (0.0%)		
Polytherapy combination (=23)								
Inducer + inhibitor	3 (13%)	1 (6.7%)	0.279		1 (6.3%)	3 (13.6%)	0.953	
Inducer + misc.	5 (21.7%)	8 (53.3%)			6 (37.5%)	7 (31.8%)		
Inhibitor + misc.	3 (13%)	1 (6.7%)			2 (12.5%)	2 (9.1%)		
2 Inducers	1 (4.3%)	0 (0%)			0 (0%)	1 (4.6%)		
2 Misc.	4 (10.5%)	3 (20%)			3 (18.7%)	4 (18.2%)		
2 Inducers + misc.	1 (4.3%)	1 (6.7%)			0 (0%)	2 (9.1%)		
Inducer + inhibitor + Misc.	6 (26.1%)	1 (6.7%)			4 (25%)	3 (13.6%)		

Menstrual cycles were considered of normal frequency if they fell within the range of 21–35 days and abnormal if they fell outside that range. Regularity was classified as normal if the variation between cycles was limited to 2–20 days over a 12-months period. Dysmenorrhea was defined as discomfort and pain during the menstrual period. Inducers: Phenytoin and Carbamazepine; Inhibitor: Valproic Acid; Misc. (Miscellaneous): Levetiracetam, Clobazam, Lamotrigine, Clonazepam, and Topiramate. The chi-square test was used to analyze categorical data, while the Mann–Whitney test was used to analyze the duration of epilepsy. SD, standard deviation; OR, odds ratio; 95% CI, 95% confidence interval; ASM, anti-seizure medication. The bold values indicates the p value is significant (<0.05).

### Reproductive hormones of WWE and WWoE

#### Reproductive hormones characteristics among WWE and WWoE

The characteristics of reproductive hormones among WWE and WWoE with and without menstrual disorders are shown in (Supplementary S1). Although the levels of reproductive hormones (FSH, LH, prolactin, and estradiol) were higher in WWE than in WWoE, the difference was not significant (*p* > 0.05). In the group without menstrual disorders, there were 42 WWE and 18 WWoE. Compared with the group with menstrual disorders, the average level of both WWE and WWoE reproductive hormones (FSH, LH, and prolactin) in the group without menstrual disorders was lower, with estradiol being the only exception. Among WWE, the average estradiol level was higher in those without menstrual disorders than in those with menstrual disorders (209.5 [509.9] and 101.72 pg/mL [123.1], respectively).

#### Reproductive hormones in WWoE and its associating factors

Supplementary S2 shows the association of reproductive hormones with demographic and clinical factors in WWoE. Hormone levels FSH, LH, and prolactin were associated with menstrual disorders in WWoE (*p* < 0.05). While estradiol level was observed to be higher in WWoE who undergoes menarche at the age <12 years old.

#### Reproductive hormones in WWE and its associating factors

In contrast to WWoE, menstrual disorders didn't show to be associated with hormone levels in WWE (Table 4). However, there was a significant association between seizure frequency and prolactin and estradiol levels in WWE (*p* < 0.001). Prolactin and estradiol levels tended to be lower with higher number of seizure frequency. FSH level was lower in WWE aged ≤ 40 years than in those aged >40 years (*p* < 0.001).

**Table 4:** The association of reproductive hormone with demographic and clinical factors in women with epilepsy.

**Variable**	**Women with epilepsy (*****N*** = **72)**							
	**FSH (mIU/ml)**		**LH (mIU/ml)**		**Prolactin (ng/ml)**		**Estradiol (pg/ml)**	
	**Mean (SD)**	***P*-value**	**Mean (SD)**	***P*-value**	**Mean (SD)**	***P*-value**	**Mean (SD)**	***P*-value**
Age (years)								
≤ 40 years old	8.74 (16.1)	**0.000**	13.48 (17)	0.15	24.93 (29.4)	0.463	188.1 (440.8)	0.327
>40 years old	38.68 (43.6)		21.25 (16.3)		18.46 (16.1)		61.46 (51.9)	
Age at onset of epilepsy		0.436		0.177		0.891		0.411
≤ 10 years old	9.82 (14.8)		10.24 (9.43)		23.1 (23)		98.37 (99)	
>10 years old	15.12 (28)		16.4 (18.8)		29.12 (3.9)		189.94 (464.1)	
Seizure type		0.127		0.815		0.081		0.557
Focal	11.1 (19)		15.1 (18)		27.12 (30.6)		183.24 (464.2)	
Generalized	21.6 (37)		13.96 (13.9)		14.03 (10.9)		117.73 (113.6)	
Controlled seizure		0.257		0.231		0.157		0.534
Yes	9.22 (18.6)		11.56 (9.8)		29.99 (36.6)		125.74 (126.7)	
No	16.27 (28.1)		16.59 (19.9)		20.37 (20.6)		188.97 (495.8)	
Frequency of seizures		0.891		0.766		**0.000**		**0.000**
1	22.57 (36.6)		18.38 (21.4)		20.05 (16.7)		123.48 (119.3)	
2	11.91 (19.1)		22 (24)		21.35 (18.1)		145.35 (150.3)	
3	13.6 (10.1)		11.6 (3)		12.43 (5.9)		100.85 (79.2)	
4	6.6 (0.5)		6.97 (1.4)		15.58 (11.4)		42.47 (18.3)	
5	4.6 (1.4)		3.2 (2)		7.19 (0.5)		68.1 (41)	
8								
25								
History of status epilepticus		0.908		0.739		0.637		0.939
Yes	12.75 (22.3)		12.86 (13.5)		28.23 (20.7)		155.10 (136.5)	
No	13.85 (25.7)		15.02 (17.5)		23.3 (28.4)		167.65 (424.1)	
Etiology of epilepsy		0.324		0.254		0.752		0.822
Structural	21.84 (37.2)		18.57 (21.5)		29.03 (38.7)		200.05 (632.7)	
Infection	7.65 (5.5)		22.45 (29.9)		16.7 (5.8)		144.83 (110.5)	
Immune	5.43 (3.4)		11.76 (9.7)		22.19 (19.8)		313.75 (97.3)	
Unknown	9.61 (14.2)		10.55 (8)		20.86 (19.8)		114.94 (130.1)	
Epilepsy syndrome		0.362		0.267		0.45		0.603
Symptomatic/cryptogenic	14.47 (26)		15.39 (17.4)		24.53 (28.2)		173.42 (418.7)	
Idiopathic	3.74 (2.4)		6.58 (5.9)		14.78 (16)		75 (23)	
Number of ASM		0.710		0.165		0.563		0.247
Monotherapy	14.90 (26.8)		17.73 (21.7)		25.85 (33.5)		106.87 (114.7)	
Polytherapy	12.67 (24.1)		12.13 (11)		22.05 (21.3)		219.47 (543.2)	
Age of menarche		0.515		0.247		0.106		0.5
<12 years old	8.21 (6.4)		8.18 (5.2)		8.96 (3.5)		74.91 (80.5)	
≥12 years old	14.42 (26.6)		15.6 (18)		25.71 (28.7)		178.19 (427.4)	
Menstrual disorders		0.06		0.315		0.249		0.542
Normal	8.29 (9.5)		12.75 (14.6)		22.21 (20.9)		209.5 (509.9)	
Abnormal								
Frequency	26.65 (48.3)		21.23 (28)		35.81 (51.6)		81.23 (120.2)	
Regularity	18.41 (28.3)		15.12 (12.2)		19.29 (17.4)		117.09 (126.8)	
Dysmenorrhea		0.071		0.141		0.934		0.188
Yes	9.33 (13.5)		12.34 (9.8)		24.07 (32.3)		217.01 (507.1)	
No	20.25 (35.6)		18.39 (23.8)		23.51 (19.1)		85.78 (83)	

Student's t-test was used to analyze data regarding demographic and clinical factors in relation to reproductive hormones. SD, standard deviation; ASM, anti-seizure medication; FSH, follicle-stimulating hormone; LH, luteinizing hormone. The bold values indicates the p value is significant (<0.05).

### Anti-seizure medication and reproductive hormones

Inducer ASMs were the most widely used ASMs (32.1%), followed by inhibitor ASMs (13%). Supplementary S3 presents the association of certain types of ASMs with reproductive hormones. There was no association between the use of ASM and reproductive hormone levels in WWE in the total cohort. However, in subgroup analysis based on the presence of menstrual disorders, some ASMs exhibited a direct relationship with certain hormones. In WWE with menstrual disorders, VPA was associated with higher estradiol levels, while lamotrigine was associated with lower FSH levels. In WWE without menstrual disorders, topiramate was associated with higher prolactin levels.

## Discussion

### Demographic and clinical characteristics

This is the first study conducted on WWE in Indonesia. We found no significant difference in age at menarche between WWoE and WWE, which are consistent with the findings of the previous (16). In contrast, there was a difference in marital status between the groups: marriage rates were lower in WWE than in WWoE. General population in Indonesia tend to be unwilling to marry people with epilepsy (PWE). Despite of good knowledge of epilepsy among general population, they were still reluctant to have deep bond with PWE. This could lead to low self-esteem and high unmarried rate among WWE (8). Similarly, a study in India also found that WWE had a lower marriage rate than the general population (9).

The clinical characteristics of the subjects included in this study were identical to those included in previous studies on epilepsy that displayed focal onset epilepsy as a common seizure type (17). The mainstream etiologies in our subjects were either structural or unknown; this is in accordance with several other studies that revealed structural (head trauma, cerebrovascular accidents) or unknown problems as the leading causes of epilepsy (18, 19). In terms of epilepsy syndromes, symptomatic/cryptogenic were more common than idiopathic types in all groups, which was also demonstrated in a Canadian study, where symptomatic, cryptogenic, and idiopathic epilepsy occurred in 44.4, 47.6, and 8% of subjects, respectively (20).

### Menstrual disorders and epilepsy

The prevalence of menstrual disorders among WWE in this study was higher than that reported in other studies. Menstrual disorders were reported in 28.8% of WWE in Poland^7^ and 39.7% of those in Nigeria (10). The irregularity of menstrual cycle in WWE could be associated with the decrease of luteal phase. This phenomenon was also seen in depression and anxiety (21). The correlation of epilepsy with menstrual disorders is thought to be due to the effect of epilepsy itself or an adverse effect of ASMs (clonazepam) on the neuroendocrine regulation of the menstrual cycle (7, 22). The prevalence of menstrual disorders in WWE who had an onset of epilepsy before menarche was higher than that in WWE with an age of epilepsy onset after menarche (7). However, there were no significant associations of age of epilepsy onset and age at menarche with menstrual disorders in this study, which is consistent with the findings of previous studies (7).

Our study showed there was no significant association between menstrual disorders in WWE and ASM. Bosak et al. (7) demonstrated no significant difference in the incidence of irregular menstruation in patients taking valproic acid, lamotrigine, levetiracetam, lacosamide, or topiramate but noted a significant association with clonazepam. Similarly, our study revealed that polytherapy with clobazam was the only ASM to have a significant relationship with menstrual disorders. Benzodiazepines, such as clonazepam and clobazam, are commonly used to treat exacerbations of hormonally affected seizures (5). The mechanism underlying the relationship between the use of benzodiazepines and menstrual disorders has not been elucidated. The number of ASMs and type of polytherapy combination were not significantly associated with dysmenorrhea and menstrual disorders; hence, duration of drug usage and drug dosage were not analyzed further.

### Dysmenorrhea in women with epilepsy

The incidence of dysmenorrhea in this study was higher in WWE (59.7%) than in WWoE (20%). Psychological factors, such as depression, stress, and anxiety, are thought to have a reciprocal relationship with the severity of dysmenorrhea and menstrual disorders. The occurrence of monthly dysmenorrhea can increase the risk of depression and vice versa. Women with dysmenorrhea are more prone to depression and anxiety (23). However this study did not include depression as a variable influencing dysmenorrhea in WWE. In addition, disturbances in the HPA axis in WWE can affect the production of reproductive hormones, which then play a role in the incidence of dysmenorrhea (24). We did not found any association between demographic and clinical factors to dysmenorrhea in WWE.

### Epilepsy and reproductive hormones

The results of this study revealed that prolactin, estradiol, FSH, and LH levels in WWE were higher than those in WWoE. FSH levels were found to be higher in the sample population aged >40 years old in both WWE and WWoE. This finding is consistent with known physiological changes, as FSH production indeed increases with age. However, after closer scrutiny, FSH levels appear significantly higher in WWE (Table 4) than in WWoE (Supplementary S2). An earlier study also indicated that WWE generally have higher levels of FSH, LH, prolactin, and estradiol than WWoE (25). Herzog et al. (26) proposed that an element of lateralization was involved in reproductive hormone disorders among WWE. Unfortunately, we did not distinguish between symptomatic epilepsy with left and right-sided foci.

In this study, a higher frequency of seizures was associated with lower prolactin and estradiol levels in the blood. The mechanisms involved remain vague, and further research is required to elucidate them. The proconvulsant nature of cortisol leads to further susceptibility and frequency of future seizures (27). The association between estrogen and seizure frequency has not been fully understood. Estradiol levels were reduced in WWE compared with those in WWoE in this study. In contrast, estrogen has both effects as pro- and anticonvulsant properties as well (28). Therefore, the lower level of estrogen could become the associated factors toward high frequency seizure in WWE and vice versa. The high number of seizure frequency could alter the pulsatile secretion of estrogen.

### ASM and reproductive hormone

The interaction between epilepsy and ASMs can cause sexual hormone imbalance (4). One study concluded that monotherapy with VPA causes a decrease in FSH and LH levels in WWE (29). An earlier study demonstrated that oxcarbazepine reduces estrogen and progesterone levels, while lamotrigine reduces estradiol levels (30). Our findings differ slightly in the sense that in WWE with menstrual disorders, VPA was associated with higher estradiol levels, while lamotrigine was associated with lower FSH levels. An *in-vitro* study demonstrated that VPA directly inhibits ovarian steroid production, namely FSH and estradiol synthesis (31); however, other studies show that VPA tends to increase estradiol activity, either through inhibition of liver enzymes or enhancement of estrogen receptors (32). Lower FSH levels have also been discovered in patients who underwent monotherapy with lamotrigine, regardless of their menstrual phase (33). In WWE without menstrual disorders, topiramate was connected to higher prolactin levels. However, the mechanism underlying the association of topiramate and prolactin is unclear due to limited data. No further association has been identified between epilepsy syndromes and etiology on reproductive hormone levels. A possible reason for the discrepancy between our study and previous studies could be the insufficient sample size and timing of retrieving blood samples.

The limitations of our study are that we did not consider the various phases of the menstrual cycle, therefore analysis of hormone levels in women without menstrual disorders could potentially be exposed to bias. Unfortunately, we did not record seizure data within 24 h of blood collection to determine the association between seizures and hormone levels, especially of prolactin. Other limitations includes absent information on lateralization of structural etiology and lack of body mass index and other clinical data (comorbidities and routine consumption of drugs) on WWoE in comparison with WWE. Due to limited time during sample collection in this pandemic era, the number of WWoE without menstrual disorders did not achieve the same level as that of the case group. Unfortunately, we could not conduct multicenter research, since we could not visit other hospital at the beginning of the pandemic era.

In conclusion, WWE were more prone to suffer from dysmenorrhea than WWoE. Unsurprisingly there was a significant difference in WWoE hormone levels, in those with and without menstrual disorders. However, this phenomenon was not observed in WWE; hence, menstrual disorders in WWE could be caused by external, non-hormonal factors. Further research opportunities are suggested with a larger sample size and multicenter to comprehend the association between dysmenorrhea and depression, as well as the focal epileptic discharge location association toward the occurrence of menstrual disorders in WWE. Moreover, additional research on menstrual disorders in WWE needs to be warranted, involving serial reproductive hormone levels examination in relation to certain phases of the menstrual cycle.

## Data availability statement

The original contributions presented in the study are included in the article/Supplementary material, further inquiries can be directed to the corresponding author.

## Ethics statement

The studies involving human participants were reviewed and approved by Faculty of Medicine Universitas Indonesia Ethics Committee and Cipto Mangunkusumo Hospital research section (Ethical review number: 1393/UN2.F1/ETIK/PPM.00.02/2019). The patients/participants provided their written informed consent to participate in this study.

## Author contributions

All authors listed have made a substantial, direct, and intellectual contribution to the work and approved it for publication.

## Funding

The authors would like to thank Universitas Indonesia for funding this research through the PUTI Grant (Contract number: NKB-1304/UN2.RST/HKP.05.00/2020).

## Conflict of interest

The authors declare that the research was conducted in the absence of any commercial or financial relationships that could be construed as a potential conflict of interest.

## Publisher's note

All claims expressed in this article are solely those of the authors and do not necessarily represent those of their affiliated organizations, or those of the publisher, the editors and the reviewers. Any product that may be evaluated in this article, or claim that may be made by its manufacturer, is not guaranteed or endorsed by the publisher.
