# Supplementary material for: Characteristics of menstrual disorders and reproductive hormones in women with epilepsy at an Indonesian national referral hospital
Source: Front Neurol. 2022 Sep 20;13:964761. doi: 10.3389/fneur.2022.964761 (PMC9531022; doi:10.3389/fneur.2022.964761)
Supplement: Supplementary file 1 [file Table_1.DOCX]

***S1.* Reproductive hormone characteristics of women with epilepsy and women without epilepsy**

| **Hormone characteristics (Mean (SD))** | **Women with epilepsy (n=72)** | **Women without epilepsy (n=50)** | **P value** |
| --- | --- | --- | --- |
|  | **N (%)** | **N (%)** |  |
| - FSH (mIU/ml) - LH (mIU/ml) - Prolactin (ng/ml) - Estradiol (pg/ml) | 13.73 (25.2)  14.78 (17)  23.85 (27.6)  166.39 (404.1) | 11.57 (19.9)  9.56 (11.2)  20.17 (42.5)  82.96 (123.9) | 0.614  0.06  0.563  0.161 |
| **Women with Menstrual Disorders (n=62)** | **Women with epilepsy (n=30)** | **Women without epilepsy (n=32)** | **P value** |
|  | **N (%)** | **N (%)** |  |
| - FSH (mIU/ml) - LH (mIU/ml) - Prolactin (ng/ml) - Estradiol (pg/ml) | 21.33 (36.4)  17.62 (19.9)  26.15 (35.2)  101.72 (123.1) | 13.46 (24.2)  11.62 (12.8)  22.89 (52.6)  98.9 (149.6) | 0.32  0.16  0.78  0.94 |
| **Women without Menstrual Disorders (n=60)** | **Women with epilepsy (n=42)** | **Women without epilepsy (n=18)** | **P value** |
|  | **N (%)** | **N (%)** |  |
| - FSH (mIU/ml) - LH (mIU/ml) - Prolactin (ng/ml) - Estradiol (pg/ml) | 8.29 (9.5)  12.75 (14.6)  22.21 (20.9)  209.5 (509.9) | 8.19 (7.5)  5.9 (6.3)  15.33 (10.3)  54.62 (46.5) | 0.968  0.061  0.19  0.206 |

SD: Standard Deviation; FSH: Follicle-Stimulating Hormone; LH: Luteinizing Hormone
