# Supplementary material for: Characteristics of menstrual disorders and reproductive hormones in women with epilepsy at an Indonesian national referral hospital
Source: Front Neurol. 2022 Sep 20;13:964761. doi: 10.3389/fneur.2022.964761 (PMC9531022; doi:10.3389/fneur.2022.964761)
Supplement: Supplementary file 2 [file Table_2.DOCX]

***S2*. The association of reproductive hormone with demographic and clinical factors in women without epilepsy**

| Variable | Women without epilepsy (N=50) | | | | | | | |
| --- | --- | --- | --- | --- | --- | --- | --- | --- |
|  | FSH (mIU/ml) | | LH (mIU/ml) | | Prolactin (ng/ml) | | Estradiol (pg/ml) | |
|  | Mean (SD) | P value | Mean (SD) | P value | Mean (SD) | P value | Mean (SD) | P value |
| Age (years)  ≤40 years old  > 40 years old | 10.43 (21.2)  15.58 (14.9) | 0.455 | 8.76 (11.2)  12.4 (11.3) | 0.346 | 22.28 (47.9)  12.69 (5.7) | 0.514 | 71.25 (61.5)  124.47 (241.9) | 0.212 |
| Age of menarche  < 12 years old  ≥ 12 years old | 10.53 (12.3)  11.7 (20.9) | 0.894 | 10.83 (9.9)  9.38 (11.5) | 0.77 | 10.96 (4.2)  21.43 (45.1) | 0.576 | 201.33 (312.7)  66.82 (62.6) | **0.011** |
| Menstrual disorder   - Normal   Abnormal | 8.19 (7.5)  13.46 (24.2) | 0.375 | 5.9 (6.3)  11.62 (12.8) | 0.083 | 15.33 (10.3)  22.9 (52.6) | 0.551 | 54.62 (46.5)  98.9 (149.6) | 0.229 |
| Menstrual disorder   - Normal   Abnormal   - Frequency - Regularity | 8.19 (7.5)  28.56 (43.6)  9.24 (14.03) | **0.048** | 5.9 (6.3)  18.01 (19.2)  9.82 (10.3) | **0.049** | 15.33 (10.3)  58.13 (110.6)  13.03 (7.3) | **0.035** | 54.62 (46.5)  152.5 (302.9)  83.89 (69.9) | 0.21 |
| Dysmenorrhea   - Yes   No | 5.87 (2.1)  12.99 (22.1) | 0.317 | 3.67 (2.6)  11.03 (12.1) | 0.063 | 15.13 (12.8)  21.43 (47.1) | 0.679 | 22.87 (17.6)  97.99 (134.4) | 0.087 |

Student’s t-test was used to analyze data regarding demographic and clinical factors in relation to reproductive hormones. SD: Standard Deviation; FSH: Follicle-Stimulating Hormone; LH: Luteinizing Hormone
