# Supplementary material for: Characteristics of menstrual disorders and reproductive hormones in women with epilepsy at an Indonesian national referral hospital
Source: Front Neurol. 2022 Sep 20;13:964761. doi: 10.3389/fneur.2022.964761 (PMC9531022; doi:10.3389/fneur.2022.964761)
Supplement: Supplementary file 3 [file Table_3.DOCX]

***S3*. The association of ASM type with hormones in WWE in general, WWE with menstrual disorders, and WWE without menstrual disorders**

| Variable |  |  | FSH (mIU/ml) | | LH (mIU/ml) | | Prolactin (ng/ml) | | Estradiol (pg/ml) | |
| --- | --- | --- | --- | --- | --- | --- | --- | --- | --- | --- |
|  | Type of ASM  (As monotherapy or polytherapy) | N (%) | Mean (SD) | P value | Mean (SD) | P value | Mean (SD) | P value | Mean (SD) | P value |
| WWE (n=72) | Phenytoin | 20 (15.3%) | 15.44 (24.4) | 0.723 | 12.74 (10.7) | 0.533 | 24.54 (26.5) | 0.896 | 283.41 (729.4) | 0.126 |
|  | Carbamazepine | 22 (16.8%) | 15.1 (29.3) | 0.762 | 11.25 (10.8) | 0.247 | 16.09 (13) | 0.114 | 99.91 (105.5) | 0.371 |
|  | Valproic acid | 17 (13%) | 8.4 (10.1) | 0.322 | 12.8 (78) | 0.578 | 14.23 (7.3) | 0.082 | 185.08 (152.5) | 0.834 |
|  | Levetiracetam | 24 (18.3%) | 13.61 (24.7) | 0.978 | 18.65 (24.4) | 0.174 | 29.75 (40.1) | 0.202 | 271.92 (685.4) | 0.127 |
|  | Clobazam | 13 (9.9%) | 22.26 (38.7) | 0.179 | 15.89 (14.7) | 0.796 | 21.26 (22.1) | 0.712 | 131.61 (131.4) | 0.746 |
|  | Lamotrigine | 16 (12.2%) | 6.91 (5) | 0.223 | 11.66 (9.2) | 0.411 | 17.53 (11.9) | 0.302 | 104.40 (120.3) | 0.507 |
|  | Topiramate | 15 (11.5%) | 7.05 (9.9) | 0.252 | 10.84 (11) | 0.318 | 29.02 (20.5) | 0.419 | 178.88 (152.9) | 0.898 |
|  | Clonazepam | 2 (1.5%) | 5.3 (1.3) | 0.635 | 6.4 (2.9) | 0.484 | 22.76 (7.1) | 0.956 | 54.75 (12.4) | 0.695 |
| WWE with menstrual disorders (n=30) | Phenytoin | 7 (12.5%) | 28.41 (38.7) | 0.566 | 19.43 (15.4) | 0.788 | 22.52 (21.5) | 0.761 | 135.45 (146.7) | 0.413 |
|  | Carbamazepine | 9 (16.1%) | 25.7 (44.4) | 0.672 | 16.14 (15.1) | 0.794 | 14.17 (9.7) | 0.229 | 111.41 (157.6) | 0.798 |
|  | Valproic acid | 7 (12.5%) | 6.44 (2.1) | 0.222 | 13.88 (6.5) | 0.579 | 10.54 (5.7) | 0.185 | 227.27 (175.5) | **0.001** |
|  | Levetiracetam | 10 (17.8%) | 19.01 (36.4) | 0.810 | 23.85 (28.1) | 0.231 | 38.17 (53.4) | 0.191 | 109.66 (144.4) | 0.819 |
|  | Clobazam | 9 (16.1%) | 27.77 (45.8) | 0.535 | 18.38 (17.4) | 0.894 | 24.64 (26.2) | 0.881 | 133.53 (150.3) | 0.397 |
|  | Lamotrigine | 7 (12.5%) | 6.41 (1.8) | **0.008** | 8.93 (5) | 0.114 | 10.84 (4.9) | 0.068 | 113.65 (180.4) | 0.263 |
|  | Topiramate | 7 (12.5%) | 5.24 (2.4) | 0.186 | 9.09 (7.4) | 0.2 | 20.81 (16.2) | 0.655 | 186.01 (164.1) | 0.057 |
|  | Clonazepam | 0 (0%) | n/a | n/a | n/a | n/a | n/a | n/a | n/a | n/a |
| WWE without menstrual disorders (n=42) | Phenytoin | 13 (17.8%) | 8.45 (6.7) | 0.944 | 9.13 (5) | 0.288 | 25.63 (29.5) | 0.483 | 363.03 (901.1) | 0.195 |
|  | Carbamazepine | 13 (17.8%) | 7.73 (6.9) | 0.802 | 7.87 (4.5) | 0.149 | 17.42 (15.1) | 0.326 | 92.83 (62.7) | 0.327 |
|  | Valproic acid | 10 (13.7%) | 9.82 (13.2) | 0.569 | 12.04 (9.1) | 0.863 | 16.81 (7.4) | 0.356 | 155.55 (135.9) | 0.706 |
|  | Levetiracetam | 14 (19.2%) | 9.75 (10.9) | 0.489 | 14.93 (21.6) | 0.497 | 27.97 (7.5) | 0.742 | 376.24 (867.3) | 0.136 |
|  | Clobazam | 4 (5.5%) | 9.87 (9.5) | 0.732 | 10.3 (1.5) | 0.729 | 13.65 (3.4) | 0.396 | 127.77 (102.8) | 0.741 |
|  | Lamotrigine | 9 (12.3%) | 7.3 (6.6) | 0.728 | 13.78 (11.3) | 0.812 | 22.73 (13.3) | 0.933 | 98.24 (69.7) | 0.467 |
|  | Topiramate | 8 (11%) | 8.63 (13.6) | 0.912 | 12.37 (13.7) | 0.937 | 36.2 (22) | **0.033** | 173.53 (155.3) | 0.828 |
|  | Clonazepam | 2 (2.7%) | 5.3 (1.2) | 0.654 | 6.4 (2.9) | 0.534 | 22.76 (7) | 0.970 | 54.75 (12.4) | 0.666 |

Student’s t-test was used to analyze data regarding ASM type in relation to reproductive hormones. SD: Standard Deviation; WWE: Women With Epilepsy; ASM: Anti-Seizure Medication; FSH: Follicle-Stimulating Hormone; LH: Luteinizing Hormone
